# Comparison of outcomes between endoscopic surgery and conventional nasal packing for epistaxis in the posterior fornix of the inferior nasal meatus

**DOI:** 10.12669/pjms.316.8340

**Published:** 2015

**Authors:** You Zou, Yu-Qin Deng, Chang-Wu Xiao, Yong-Gang Kong, Yu Xu, Ze-Zhang Tao, Shi-Ming Chen

**Affiliations:** 1 You Zou, Department of Otolaryngology Head and Neck Surgery, Renmin Hospital of Wuhan University, Wuhan, China; 2 Yu-Qin Deng, Department of Otolaryngology Head and Neck Surgery, Renmin Hospital of Wuhan University, Wuhan, China; 3 Chang-Wu Xiao, Department of Otolaryngology Head and Neck Surgery, Renmin Hospital of Wuhan University, Wuhan, China; 4 Yong-Gang Kong, Department of Otolaryngology Head and Neck Surgery, Renmin Hospital of Wuhan University, Wuhan, China; 5 Yu Xu, Department of Otolaryngology Head and Neck Surgery, Renmin Hospital of Wuhan University, Wuhan, China; 6 Ze-Zhang Tao, Department of Otolaryngology Head and Neck Surgery, Renmin Hospital of Wuhan University, Wuhan, China; 7 Shi-Ming Chen, Department of Otolaryngology Head and Neck Surgery, Renmin Hospital of Wuhan University, Wuhan, China

**Keywords:** Posterior fornix, Inferior nasal meatus, Epistaxis, Endoscopy, Electrocautery

## Abstract

**Objective:**

To investigate the clinical features of epistaxis in the posterior fornix of the inferior nasal meatus and compare the treatment outcomes of endoscopic surgery and conventional nasal packing for this intractable form of epistaxis.

**Methods:**

Between August 2011 and August 2014, the medical records of 53 adult patients with idiopathic epistaxis in the posterior fornix of the inferior nasal meatus diagnosed by nasal endoscopy were obtained from our department. Of these, 38 patients underwent endoscopic surgery (surgery group) and 15 received a nasal pack (packing group). The patients’ background characteristics, incidence of re-bleeding, extent of discomfort after treatment as assessed using a 10-point visual analogue scale (VAS) and incidence of nasal cavity adhesion after treatment were analysed.

**Results:**

There were no significant differences in background characteristics between the two groups. The incidence of re-bleeding (0/38 vs. 4/15, surgery vs. control, *P* = 0.001), VAS score for discomfort (2.4 ± 1.4 vs. 7.6 ± 1.0, surgery vs. control, *P* = 0.001) and incidence of nasal cavity adhesion after treatment (2/38 vs. 7/15, surgery vs. control, *P* = 0.007) were significantly lower in the surgery group than in the packing group.

**Conclusion:**

Endoscopic surgery is superior to conventional nasal packing for the management of epistaxis in the posterior fornix of the inferior nasal meatus. During surgery, it is crucial to expose the bleeding sites by shifting the inferior turbinate inward by fracture.

## INTRODUCTION

Epistaxis is one of the most common otolaryngological emergencies. Although most cases of epistaxis can be managed by compression of the nostrils, packing of the affected nostrils, angiotonics and sedatives^1^, the treatment of intractable epistaxis using conventional methods remains a challenge because the bleeding points are occult and deep.^2^ Posterior epistaxis accounts for 5–10% of all intractable epistaxis cases, resulting in massive haemorrhage and requiring more aggressive measures for haemostasis.^3^

The effectiveness of conventional packing of the anterior or posterior nares for the control of epistaxis in the posterior fornix of the inferior nasal meatus (a type of intractable posterior epistaxis) is reportedly unsatisfactory.^4^ The management of posterior epistaxis is quite challenging for physicians and may be extremely painful for the patient. Most of the bleeding sites are localized at the posterior end of the inferior nasal meatus, where the posterolateral nasal arteries are localized.^5^ The indications of surgery or conventional nasal packing were previously generalized.^6^ Although nasal packing is more simple procedure compared with surgery, it may not compress all the bleeding sites.^7^ On the other hand, surgery has been reported to be effective in the management of this condition.^1^ Nevertheless, some patients may choose packing because of reluctance to undergo surgery. In this study, we investigated the clinical features of epistaxis in the posterior fornix of the inferior nasal meatus and compared the outcomes of endoscopic surgery and conventional nasal packing for this intractable form of epistaxis.

## METHODS

### Patients

The study population comprised 53 consecutive adult patients with epistaxis in the posterior fornix of the inferior nasal meatus who underwent endoscopic surgery or received a nasal pack at our department between August 2011 and August 2014. The condition was diagnosed by nasal endoscopy in all 53 patients, 15 of whom received a nasal pack (packing group) and 38 of whom underwent endoscopic surgery (surgery group).

The exclusion criteria were as follows: age < 20 years, bleeding from sites other than the posterior fornix of the inferior nasal meatus.^8^

### Treatment

### Nasal packing

Before nasal packing, the patient was asked to refrain from talking. Subsequently, the blood clot, capillary haemorrhage and arterial hemorrhage were removed using a suction apparatus. Following the induction of local anaesthesia, a piece of haemostatic cotton (10.0 × 1.0 × 2.0 cm, Shending Industruy Co., Ltd., Shanghai, China) was gently placed into the nasal cavity and removed 48–72 h later.

### Surgical intervention

**Fig.1:**
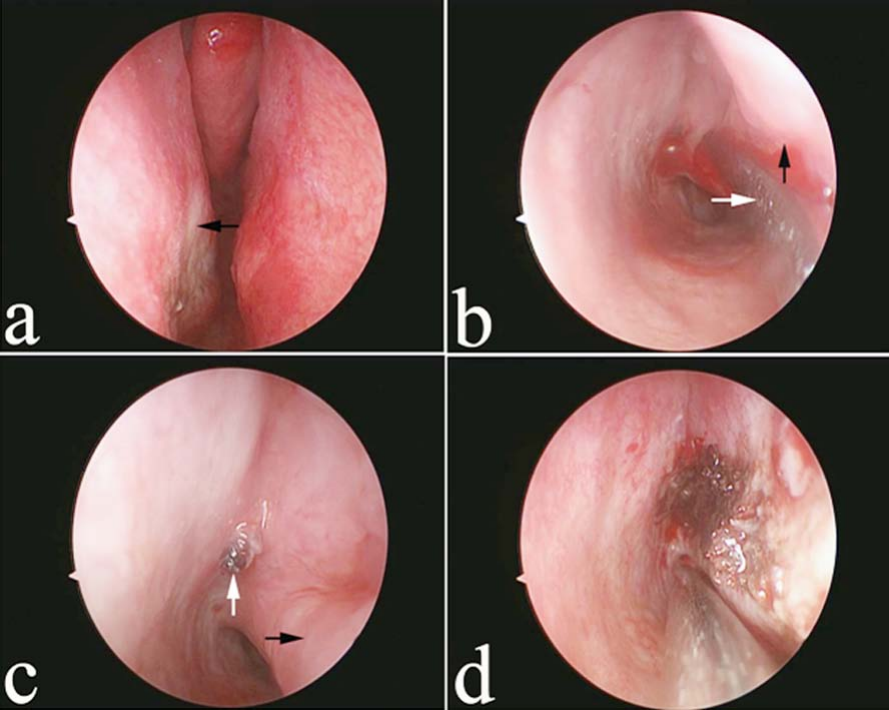
Surgical intervention. The bleeding points are not located in the olfactory fissure, sphenoethmoidal recess, or middle nasal meatus, as observed during nasal endoscopy (black arrow indicates the right middle turbinate and white arrow indicates the right inferior turbinate).The inferior nasal concha is shifted by fracture to broaden the inferior nasal meatus (black arrow indicates the strip apparatus and white arrow indicates the right inferior turbinate).The cut end of the right posterolateral nasal artery located in the posterior fornix of the inferior nasal meatus (white arrow indicates the right inferior turbinate and black arrow indicates the cut end of the artery).The bleeding sites are treated using electrocautery until no active bleeding can be observed.

The interior nasal cavity was explored and bipolar electric hemostat performed under general anaesthesia in three patients with recurrent epistaxis and poor tolerance. In the remaining patients, the procedures were performed under local anaesthesia. Because the majority of patients had received multiple nasal packs and/or had undergone electrocautery, they were first counselled to eliminate or relieve potential fear. During surgery, the patient was placed in the supine position, the nasal pack was removed under endoscopic guidance and the bleeding points, particularly those located in the olfactory fissure, sphenoethmoidal recess and middle nasal meatus, were clearly examined from a cranio-caudal perspective after the application of topical anaesthesia using tetracaine and oxymetazoline hydrochloride spray (Fig.1a). If no significant bleeding points were detected and accumulation of blood in the posterior nares was observed, intractable posterior epistaxis was suspected. For these patients, gauze containing tetracaine and oxymetazoline hydrochloride spray was inserted into the inferior nasal meatus until it reached the posterior part and compression was performed. Subsequently, the gauze was removed and the inferior nasal concha was shifted by fracture to broaden the inferior nasal meatus (Fig.1b). Subsequently, the nasal endoscope was inserted, the mucous membrane was examined and a suction apparatus was used to remove the bloody secretions. With regard to the primary bleeding sites, significant active and even pulsating bleeding was noticed after suction (Fig.1c). The bleeding sites were then treated using bipolar electric hemostat until blanching of the peripheral mucous membrane and no active bleeding were observed (Fig.1d), followed by the insertion of absorbable cotton. After the inferior turbinate was shifted by fracture, to prevent nasal cavity adhesion and nasal obstruction after treatment, the fracture was repositioned and absorbable cotton was placed over the eroded mucous membrane.

### Outcome measures

The patients’ background characteristics, failure of therapy (defined as significant posterior re-bleeding necessitating further treatment), extent of discomfort after treatment and incidence of nasal cavity adhesion after treatment were evaluated and compared between groups. The patients’ background characteristics included gender, age, history of nasal bleeding, blood pressure (BP), haemoglobin level, hypertension, current anticoagulant use, current dialysis therapy, history of cerebral infarction and presence of cardiac disorder, diabetes mellitus, malignant tumor(s), any hepatic disorder, bronchial asthma, any thyroid disorder and/or hyperlipidaemia.^8^ Patients were followed-up at regular intervals by telephone interview until August 2014. The patients were interviewed by qualified staff and were asked several questions to establish the following VAS points after treatment. Using a 10-point visual analogue scale (VAS), the patients were asked to rank the extent of discomfort after treatment (0, no discomfort; 9, unbearable).^9^ We retrospectively reviewed nasal cavity adhesion of all patients who were followed up for 6 months after treatment.

### Statistical analysis

Continuous variables are expressed as means ± standard deviations (SDs). Student’s t-test or the Mann–Whitney U test was performed for inter-group comparisons. Intra-group comparisons were conducted using Fisher’s exact test or the χ-square test. All statistical analyses were performed using SPSS 17.0 software (SPSS, Chicago, IL). A *P*-value of <0.05 was considered statistically significant.

## RESULTS

The background factors of patients in both groups are shown in Table-I. There was no significant difference in gender (*P* = 0.698) age (49.5 ± 12.7 vs. 53.1 ± 10.9 years, surgery vs. packing, *P* = 0.340) systolic BP (134.7 ± 24.5 vs. 135.5 ± 25.7 mmHg, surgery vs. packing, *P* = 0.915) and diastolic BP (96.4 ± 3.7 vs. 96.2 ± 3.8 mmHg, surgery vs. packing, *P* = 0.883), hemoglobin level (122.7 ± 20.8 vs. 121.6 ± 21.4 g/L, surgery vs. packing, *P* = 0.646) and any of the other background characteristics between the two groups.

**Table-I:** Characteristics of patients before surgery (surgery group) or nasal packing (packing group).

	Number	Surgery group	Packing group	P
Total	53	38	15	
Gender (female: male)	26:27	18:20	8:7	0.698
Age (years) (mean±SD)		49.5±12.7	53.1±10.9	0.340
History of epistaxis (absence: presence)	46:7	33:5	13:2	0.990
SBP (mmHg)		134.7±24.5	135.5±25.7	0.915
DBP (mmHg)		96.4±3.7	96.2±3.8	0.883
Hemoglobin (g/L)		122.7±20.8	21.6±21.4	0.646
Anticoagulant medication (absence: presence)	50:3	36:2	14:1	0.844
History of hypertension (absence: presence)	44:9	32:6	12:3	0.716
Receiving dialysis (absence: presence)	53:0	38:0	15:0	1.000
Cerebral infarction (absence: presence)	50:3	36:2	14:1	0.844
Cardiac disorder (absence: presence)	43:10	31:7	12:3	0.896
Diabetes mellitus (absence: presence)	48:5	36:2	12:3	0.101
Malignant tumor (absence: presence)	53:0	38:0	15:0	1.000
Hepatic disorder (absence: presence)	53:0	38:0	15:0	1.000
Bronchial asthma (absence: presence)	53:0	38:0	15:0	1.000
Thyroid disorder (absence: presence)	53:0	38:0	15:0	1.000
Shock(absence: presence)	52:1	38:0	14:1	0.111
Hyperlipidemia (absence: presence)	47:6	34:4	13:2	0.774

Compared with the packing group, there were no significant differences between the surgery and packing groups.

The incidence of re-bleeding was significantly lower in the surgery group (0/38) than in the packing group (4/15; *P* = 0.001; Table-II). Re-bleeding occurred within 48 and 24 hour after nasal pack removal in three and one patient, respectively. All patients with re-bleeding underwent endoscopic surgery with successful achievement of haemostasis.

**Table-II:** Incidence of re-bleeding, visual analogue scale score for the extent of discomfort and incidence of nasal cavity adhesion in the surgery and nasal packing group.

	Surgery group	Packing group	P
Re-bleeding	0	4	0.001
Discomfort after the treatment	2.4±1.4	7.6±1.0	0.001
Adhesion of nasal cavity	2	7	0.007

The re-bleeding, extent of discomfort and incidence of nasal cavity adhesion were significantly lower in the surgery group than in the packing group.

Discomfort in both groups was primarily attributed to nasal obstruction, nasal secretion, epistaxis and headache. The VAS score for extent of discomfort significantly differed between groups, ranging from 1 to 5 in the surgery group (2.4 ± 1.4) and 6 to 9 in the packing group (7.6 ± 1.0; *P* = 0.001; Table-II).

Nasal cavity adhesion can lead to nasal obstruction and, occasionally, sinusitis. Therefore, it is an important target of treatment. The incidence of nasal cavity adhesion was significantly lower in the surgery group (2/38) than in the packing group (7/15; *P* = 0.007; Table-II).

## DISCUSSION

This study evaluated the outcomes of endoscopic surgery and conventional nasal packing for epistaxis in the posterior fornix of the inferior nasal meatus. The failure rate, extent of discomfort and incidence of nasal cavity adhesion were significantly lower in the surgery group than in the packing group, indicating that endoscopic surgery was superior to conventional nasal packing.

In all 53 patients, the bleeding sites were mostly localized in the posterior nasal dome of the inferior nasal meatus, with the large branches of the posterolateral nasal arteries distributed around the external lateral wall.^10^ Intermittent bleeding was observed before surgery or nasal packing, with blood effusion from the anterior naris, pharynx oralis, contralateral nasal cavity, affected eyes and the external acoustic meatus; these are the typical clinical features of epistaxis in the posterior fornix of the inferior nasal meatus. This form of epistaxis is characterized by instant bleeding and haemostasis, with vague symptoms in the early stage.^11^ Intractable posterior epistaxis can be divided into nasal bleeding or bleeding from the pharynx oralis.^12^ Patients with epistaxis in the pharynx oralis who are misdiagnosed and receive an unnecessary nasal pack may present with blood gushing from the oral cavity and/or bleeding from the contralateral nasal cavity, with occasional bleeding from the eyes (nasolacrimal duct reflux). In addition, blood may gush from the pharynx oralis or contralateral nasal cavity in patients who receive a post-nasal pack. In rare circumstances, patients may exhibit perforation of the tympanic membrane with blood gushing from the external acoustic meatus.^13^

The primary goal of treatment for epistaxis is the identification of the bleeding sites, followed by immediate and effective haemostasis.^14^ Patients with epistaxis in the posterior fornix of the inferior nasal meatus should receive immediate treatment after pre-operative preparation, which include fluid infusion, anti-inflammatory therapy and, in some cases, anti-shock therapy. Unexpected events may occasionally occur during nasal endoscopy, packing and/or electrocautery because of anxiety, mental stress and poor tolerance to severe pain. To resolve these issues, careful nasal cavity exploration and electrocautery were recommended under nasal endoscopy in the operating room on the basis of our clinical experience. In our study, recurrent bleeding (six times) occurred in one patient before surgery, with an approximate total bleeding volume of 1400 mL. Previously, nasal and post-nasal packs were placed together with electrocautery (in quadruplicate); however, intermittent bleeding and even shock were observed. Therefore, blood transfusion, fluid infusion and antibiotic therapy were administered. Finally, after stabilization of the patient’s condition, surgery was performed under general anaesthesia. The bleeding site was found in the right posterior fornix of the inferior nasal meatus and haemostasis was achieved.

Identification of the bleeding sites is crucial for successful outcomes of surgery. In our study, extensive efforts were made to identify potential bleeding sites in the olfactory fissure, sphenoethmoidal recess and middle nasal meatus; the results indicated no active bleeding. Therefore, the inferior nasal concha was shifted in the direction of the nasal septum by fracture and the inferior nasal meatus was broadened. During nasal endoscopy, fresh blood and occasional pulsating bleeding were observed. In one patient with suspected epistaxis in the posterior fornix of the inferior nasal meatus, bleeding could not be controlled after nasal cavity exploration and electrocautery. Subsequently, the patient was transferred to our department, and nasal endoscopy indicated bleeding in the posterior nares despite extensive cauterization in the affected inferior nasal concha and the anterior mucous membrane of the inferior nasal meatus. On shifting of the inferior nasal concha by fracture, bleeding induced by arterial rupture was observed in the posterior fornix of the inferior nasal meatus and was controlled using bipolar coagulation.

Most cases of epistaxis in our study could be managed by electrocautery if the bleeding point was localized. In addition, special attention was paid to prevent capillary haemorrhage. Post-operative bleeding and nasal cavity adhesion in the vesicated mucous membrane of the nasal cavity and affected region were prevented by the placement of absorbable cotton, which was removed after 1 or 2 weeks.

### Limitations of the study

There are some limitations in our study. The patients was only from our department, which may not have enough statistical power to effectiveness. In the later research, we will study patients in different regions and countries to increase the number of patients.

## CONCLUSION

Endoscopic surgery can be effective for the treatment of intractable epistaxis in the posterior fornix of the inferior nasal meatus, and shifting of the inferior nasal concha by fracture and localization of the bleeding points are crucial for successful surgical outcomes. Immediate surgery is recommended for patients with recurrent and massive haemorrhage.
